# Comparison of Intraocular Pressure and Anterior Chamber Angle Changes between Pilocarpine and Laser Peripheral Iridotomy

**DOI:** 10.5005/jp-journals-10078-1245

**Published:** 2019

**Authors:** Ardiella Yunard, Virna D Oktariana, Widya Artini, Joedo Prihartono

**Affiliations:** 1–3 Department of Ophthalmology, Faculty of Medicine, Universitas Indonesia, Cipto Mangunkusumo Hospital, Jakarta, Indonesia; 4 Department of Community Medicine, Faculty of Medicine, Universitas Indonesia, Cipto Mangunkusumo Hospital, Jakarta, Indonesia

**Keywords:** Anterior chamber angle, Clinical trial, Intraocular pressure, Laser peripheral iridotomy, Pilocarpine

## Abstract

**Aim:**

To compare intraocular pressure and anterior chamber angle changes between pilocarpine and laser peripheral iridotomy in primary angle closure.

**Materials and methods:**

In this clinical trial study, 34 eyes of 29 patients with primary angle-closure were prospectively enrolled between November 2015 and February 2016. Intraocular pressure and anterior segment optical coherence tomography were performed at three separate times: on the initial conditions, 3–5 days of administration of topical pilocarpine 2%, and 1 week after laser iridotomy. Anterior chamber angle parameters were the angle opening distance (AOD) and trabecular–iris space area (TISA).

**Results:**

The intraocular pressure reduction following pilocarpine administration was significant compared to laser iridotomy: 3.9 mm Hg (−32.5 to 0.20) vs 1.8 mm Hg (−33.5 to 2.30) (*p* = 0.002). Meanwhile, the increment of angle parameters following laser iridotomy was significant compared to pilocarpine. The AOD750 increment of both nasal and temporal quadrant following laser iridotomy was significant compared to pilocarpine: 0.13 mm (−0.27 to 0.28) vs 0.05 mm (−0.35 to 0.29) (*p* = 0.003) and 0.12 mm (−0.10 to 0.34) vs 0.04 mm (−0.27 to 0.19) (*p* = 0.002), respectively. The TISA750 increment of both nasal and temporal quadrant following laser iridotomy was also significant compared to pilocarpine: 0.05 mm^2^ (−0.06 to 0.20) vs 0.02 mm^2^ (−0.12 to 0.13) (*p* = 0.023) and 0.04 mm^2^ (−0.04 to 0.17) vs 0.01 mm^2^ (−0.14 to 0.18) (*p* = 0.012), respectively.

**Conclusion:**

Laser peripheral iridotomy widens the angle greater than topical pilocarpine, but topical pilocarpine lowers the intraocular pressure greater than laser iridotomy. These data suggest that pilocarpine has another mechanism to decrease the intraocular pressure in primary angle-closure, besides widening the angle.

**How to cite this article:**

Yunard A, Oktariana VD, *et al.* Comparison of Intraocular Pressure and Anterior Chamber Angle Changes between Pilocarpine and Laser Peripheral Iridotomy. J Curr Glaucoma Pract 2019;13(1):32–36.

## INTRODUCTION

Visual impairment in primary angle closure glaucoma is 2–3 times more common compared to primary open angle glaucoma despite the less prevalence of primary angle-closure glaucoma compared to primary open angle glaucoma.^1,2^ Acute attack of angle closure glaucoma causes blindness in 10% of the total cases.^2,3^ Quigley et al.^4^ predicted the number of patients with bilateral blindness due to angle closure glaucoma in 2020 would reach 5.3 million.

Relative pupillary block is the main mechanism of aqueous flow obstruction in angle closure glaucoma patients. In pupillary block, there is an aqueous flow obstruction from posterior to anterior chamber through pupil, causing higher pressure in posterior chamber than the anterior chamber. This pressure gradient pushes peripheral iris to anterior, closing the anterior chamber angle.^5,6^

Anterior chamber angle evaluation with gonioscopy is the gold standard until now, but it has several limitations such as subjective result, dependency on examiner expertise, anatomical distortion due to pressure effect, and difficulty in quantitative measurement.^2,7–9^

Anterior segment optical coherence tomography (ASOCT) is an imaging technique which is able to give cross-sectional images of the anterior segment with high resolution. The advantages of ASOCT are faster and easier procedure, more comfortable for the patients with no contact involved, less anatomical distortion caused by contact pressure, as well as quantitative and more objective results.^2,8,10^

Therapeutic principle in angle closure is by removing pupillary block. Laser peripheral iridotomy (LPI) is the treatment of choice by making a hole in iris, allowing aqueous flow shunt from the posterior to anterior chamber and causing convex iris configuration to be more flattened and opening anterior chamber angle.^2,8,11,12^

The use of miotic agents such as pilocarpine in shallow angle is still currently debated. The rationale for pilocarpine use is to pull peripheral iris away from trabecular meshwork to open the anterior chamber angle. However, pilocarpine can also cause closure of the angle through the anterior shifting of iris–lens diaphragm due to ciliary muscle contraction and worsens pupillary block.^13^ According to Edwards et al.,^14^ pilocarpine gives protective effect to the fellow eye in patients with acute attack. Within a mean period of 5 days after acute attack, the probability of acute attack on the fellow eye is 3.3% when using pilocarpine eye drops. Meanwhile, if it is not given any therapy, the probability of acute attack in the fellow eye is 20%.^14^ Pilocarpine indication nowadays is as a prophylaxis of acute attack in angle closure glaucoma before iridotomy.^9,15^

Laser peripheral iridotomy is the standard first line therapy for angle closure. However, in Indonesia as a developing country, there are many fringe areas without laser facility. Pilocarpine eye drops is still a temporary therapy of choice while waiting for laser peripheral iridotomy to be available.^2,6,16^

The objective of this study was to compare IOP and anterior chamber angle changes between topical pilocarpine and laser peripheral iridotomy.

## MATERIALS AND METHODS

We conducted this interventional clinical trial in Cipto Mangunkusumo—Kirana Hospital from November 2015 until February 2016. Ethical review clearance test and informed consent were obtained.

### Study Participants

Patients with primary angle-closure were included in this study. If the patient was administered topical pilocarpine before the study, wash-out procedure for 48 hours was performed. Individuals excluded if they had cup disc ratio (CDR) ≥0.8, previous intraocular laser or surgery, hazy cornea, and any form of secondary angle closure.

Primary angle-closure included in this study were patients diagnosed as primary angle closure suspect (PACS), primary angle closure (PAC), and primary angle-closure glaucoma (PACG). The diagnosis of PACS was made in eyes with iris and trabecular meshwork contact ≥180° on gonioscopy, IOP ≤ 21 mm Hg, and without presence of peripheral anterior synechiae (PAS). Primary angle closure (PAC) was diagnosed as iris and trabecular meshwork contact ≥180° on gonioscopy, with either IOP > 21 mm Hg, and/or with the presence of peripheral anterior synechiae (PAS). Meanwhile, the diagnosis of PACG was made in eyes with iris and trabecular meshwork contact ≥180° on gonioscopy plus evidence of glaucomatous damage to optic disc and visual field.

The patients were recruited consecutively. If both eyes met the criteria, both were included, with a 1 week interlude between them.

Each patient underwent a standardized ophthalmic examination that included fundus examination, visual acuity determination with a Snellen chart, IOP measurement using Goldmann applanation tonometry, gonioscopy with 4-mirror lens, and ASOCT examination (Cirrus; Carl Zeiss). Examinations were performed at three separate times: at initial baseline visit, 3–5 days following topical pilocarpine administration, and 1 week following laser peripheral iridotomy.

### Topical Pilocarpin (2%)

The patients were prescribed pilocarpine 2% eyedrops after initial baseline measurement. They were instructed to administer the eyedrops 4 times a day for 3–5 days. Following administration of topical pilocarpine, the patients underwent measurements before laser procedure.

### Laser Peripheral Iridotomy (LPI)

Laser peripheral iridotomy was performed in the superior region of the iris (10-o'clock to 2-o'clock position) using topical anesthesia with sequential argon and Nd:Yag lasers. It was performed by two glaucoma consultants (VD and WA) under miotic condition of the pupil following administration of topical pilocarpine 2% for 3–5 days. Prednisolone acetate 1% eyedrops were prescribed 4 times a day for a week after procedure. Evaluation was done a week after LPI.

### Measurements of Anterior Chamber Angle

Using Cirrus OCT, two anterior chamber angle parameters (AOD750 and TISA750) were measured. It was performed by one operator for all cases. Angle opening distance was calculated as the perpendicular distance measured from the trabecular meshwork at 750 μm anterior to the scleral spur to the anterior iris surface (AOD750). Trabecular–iris space area was calculated as an area bounded anteriorly by the AOD750, posteriorly by a line drawn from the scleral spur perpendicular to the plane of the inner scleral wall to the opposing iris, superiorly by the inner corneoscleral wall, and inferiorly by the iris surface (TISA750). Measurements were taken at both nasal and temporal quadrants.

### Statistical Analysis

The primary outcome of this study was anterior chamber angle parameters and IOP. Statistical analysis was done using SPSS 21.0. Wilcoxon test was used to compare the change after pilocarpine and laser peripheral iridotomy.

## RESULTS

A total of 34 eyes with angle closure were recruited for the study. No participant had ever dropped out.

Table 1 summarizes the demographic and clinical data of the participants including age, gender, visual acuity, diagnosis group, and amount of PAS in PAC and PACG group. The mean (SD) age was 58.1 (8.9) years (age range, 46–80 years). Most of the participants were female.

The baseline IOP and angle parameters were not significantly different among diagnosis groups (Table 2). The IOP reduction by pilocarpine was significantly greater than LPI (*p* = 0.002). Meanwhile, changes of anterior chamber angle post-LPI were significantly greater than post-pilocarpine (Table 3).

Primary angle closure glaucoma had significantly the greatest IOP reduction among other diagnosis group, both post-pilocarpine (*p* = 0.048) (Table 4) and post-LPI (*p* = 0.002) (Table 5). There were no significant differences of anterior chamber angle changes post-pilocarpine and post-LPI among diagnosis groups (Tables 4 and 5).

**Table 1:** Baseline characteristics

*Variable*	
Age (year)	58.1 ± 8.9
Gender	
Male	8
Female	21
BCVA	logMAR 0 (logMAR 0 − logMAR 0.60)
Diagnosis	
PACS	16
PAC	11
PACG	7
PAS amount (clock hour)	
PAC	0–9
PACG	3–11

**Table 2:** Baseline intraocular pressure and angle parameters among groups

*Variable*	*PACS*	*PAC*	*PACG*	p^*^
IOP (mm Hg)	14.77 ± 3.71	17.16 ± 3.29	18.70 (16.00–50.50)	0.054
AOD750 (mm) nasal	0.238 ± 0.16	0.214 ± 0.17	0.130 (0.08–0.65)	0.635
AOD750 (mm) temporal	0.260 ± 0.15	0.246 ± 0.16	0.140 (0.10–0.47)	0.477
TISA750 (mm^2^) nasal	0.123 ± 0.07	0.084 ± 0.07	0.05 (0.03–0.21)	0.327
TISA750 (mm^2^) temporal	0.141 ± 0.06	0.110 ± 0.07	0.07 (0.02–0.21)	0.087

* Kruskal–Wallis test

**Table 3:** Comparison of IOP and anterior chamber angle changes between pilocarpine and laser peripheral iridotomy

*Variable*	*Pilocarpine*		*Laser peripheral iridotomy*		p*
	*Median*	*Range*	*Median*	*Range*	
ΔIOP	−3.90	−32.5 to 0.20	−1.80	−33.5 to 2.30	0.002
ΔAOD750 nasal	0.05	−0.35 to 0.29	0.13	−0.27 to 0.28	0.003
ΔAOD750 temporal	0.04	−0.27 to 0.19	0.12	−0.10 to 0.34	0.002
ΔTISA750 nasal	0.02	−0.12 to 0.13	0.05	−0.06 to 0.20	0.023
ΔTISA750 temporal	0.01	−0.14 to 0.18	0.04	−0.04 to 0.17	0.012

Wilcoxon rank

**Table 4:** Comparison of IOP and anterior segment changes after pilocarpine among diagnosis

*Variable*	*Δbaseline-pilocarpine*			*p*
	*PACS*	*PAC*	*PACG*	
ΔIOP (mm Hg)	−2.80 (−10.00–0.70)	−4.08 ± 3.2	−12.17 ± 10.67	0.048^a^
ΔAOD750 (mm) nasal	0.042 ± 0.11	0.03 (−0.25–0.13)	0.07 (−0.35–0.19)	0.803^a^
ΔAOD750 (mm) temporal	0.007 ± 0.11	−0.016 ± 0.12	0.076 ± 0.08	0.271^a^
ΔTISA750 (mm^2^) nasal	0.022 ± 0.05	0.02 (−0.12–0.40)	0.026 ± 0.06	0.680^a^
ΔTISA750 (mm^2^) temporal	0.02 ± 0.05	−0.01 ± 0.05	0.04 ± 0.07	0.219^b^

^a^ Kruskal–Wallis

^b^ ANOVA

**Table 5:** Comparison of IOP and anterior segment changes after laser peripheral iridotomy among diagnosis

*Variable*	*Δbaseline-laser peripheral iridotomy*			*p*
	*PACS*	*PAC*	*PACG*	
ΔIOP (mm Hg)	−1.11 ± 1.87	−2.88 ± 3.13	−7.40 (−33.50 to −2.00)	0.002^a^
ΔAOD750 (mm) nasal	0.119 ± 0.12	0.14 (−0.01 to 0.17)	0.021 ± 0.17	0.205^a^
ΔAOD750 (mm temporal	0.139 ± 0.14	0.101 ± 0.11	0.032 ± 0.04	0.442^b^
ΔTISA750 (mm^2^) nasal	0.049 ± 0.06	0.05 ± 0.03	0.017 ± 0.06	0.345^b^
ΔTISA750 (mm^2^) temporal	0.060 ± 0.06	0.039 ± 0.05	0.00 (−0.30 to 0.15)	0.268^a^

^a^ Kruskal–Wallis

^b^ ANOVA

## DISCUSSION

Since pupillary block is the main mechanism causing closed angle, the LPI and pilocarpine 2% eyedrops are able to eliminate pupillary block through different mechanisms to open the angle.

Comparison of anterior chamber angle changes between pilocarpine 2% eyedrops and LPI was significantly different. Laser peripheral iridotomy widened the angle more than pilocarpine. Similar to this research, Talajic et al.^9^ using pentacam observed that angle changes by LPI were wider and statistically significant compared to pilocarpine.

The change in angle parameters post-pilocarpine was not different among diagnosis groups. This was possible because pilocarpine works through two different mechanisms, pupillary sphincter muscle contraction causing miosis and pulling peripheral iris, and ciliary muscle contraction which causes lens accommodation and iridolenticular shifts to the anterior. The results of these two mechanisms determined the angle alteration.^9,17–19^ Besides that, there was a report that age affects angle alterations by pilocarpine. Ciliary muscle response on pilocarpine decreased in accordance with increasing age.^15^

The greatest change in angle parameters of each group post-LPI was in the PACS group, followed by PAC, and the least was PAC, although statistically not significant. This was similar to a study by Han et al.^20^ who observed a greater change in PAC than PACG, although not statistically significant. Another study by Ang et al.,^10^ divided the group into 2, PACS and PAC in 1 group with PACG in another group. There was no difference of angle parameter change post-LPI between the two groups. The greatest change in angle parameters post-LPI was observed in the PACS group in this study because in PACS the iridotrabecular apposition was still reversible. Meanwhile, there was already synechiae in PAC and PACG, making the angle more difficult to be opened. There were also some patients in PAC (5 patients) and PACG group (4 patients) in this study had PAS at the superior quadrant, which was the site of LPI. In PACG, besides synechiae, there was also more damage of trabecular meshwork and optical nerve. Therefore, the angle change in PACG post-LPI was the smallest among other diagnosis groups.^21^

Intraocular pressure post-pilocarpine and LPI in this study showed a significant reduction. Administration of topical pilocarpine 2% eyedrops gave statistically significant greater IOP reduction compared to LPI. The greater IOP reduction by pilocarpine, which was not in accordance with the angle changes compared to LPI implied other IOP reduction mechanism of pilocarpine. Pilocarpine, a parasympathomimetic agent pulls scleral spur posteriorly, opening intertrabecular space and Schlemm's canal, improving aqueous outflow.^17,22,23^ This mechanism gave greater IOP reduction, aside from opening the angle.

Intraocular pressure change post-pilocarpine 2% eyedrops and LPI showed the greatest reduction in PACG, followed by PAC and PACS. This was caused by the higher initial IOP of PACG compared to PAC and PACS. In the PACS group, despite having an initial IOP value within the normal range, it still showed a reduction of IOP either post-pilocarpine administration or LPI. This suggested that both pilocarpine 2% eyedrops and LPI induced an IOP reduction effect. However, it should be noted that in the PACG or PAC group with initial IOP > 21 mm Hg, antiglaucoma medicine was administered since the beginning of this study until post-LPI measurements were performed. None of the participants in this study got rescue treatment within a 1 week post-LPI follow-up phase.

The strengths of this study were no dropped-out participants, and was the first study comparing the effect of topical pilocarpine and LPI on IOP and anterior chamber angle changes. The limitation of this study was that angle parameters assessed using AS-OCT were only at the nasal and temporal quadrant.

## CONCLUSION

Both laser peripheral iridotomy and pilocarpine widen the angle and reduce the intraocular pressure of primary angle closure groups. Their effect was decreased in accordance with the severity of the disease. Primary angle-closure suspect showed the greatest result, followed by primary angle closure, and primary angle closure glaucoma was the least. Laser peripheral iridotomy widen the angle more than pilocarpine, meanwhile pilocarpine reduces the intraocular pressure more than laser peripheral iridotomy. This suggests pilocarpine has other mechanism to reduce intraocular pressure in primary angle-closure besides widening the angle.

## CLINICAL SIGNIFICANCE

By assessing the changes in intraocular pressure and anterior chamber angle after pilocarpine administration and laser iridotomy, we can identify the mechanism and response of the eye to both treatments.

## MANUFACTURER NAME

Pilocarpine 2%: Cendo Carpine 2% (Cendo Pharmaceutical, Kota Bandung, Jawa Barat 40258, Indonesia).

## PATIENT CONSENT FORM

Ethics committee approval was obtained.
